# Neighborhood Environment and Affective Walking Experience: Cluster Analysis Results of a Virtual-Environment-Based Conjoint Experiment

**DOI:** 10.3390/ijerph20021396

**Published:** 2023-01-12

**Authors:** Bojing Liao, Xiang Li

**Affiliations:** 1 Institute of Creativity and Innovation, Xiamen University, Xiamen 361005, China; 2 School of Architecture and Civil Engineering, Xiamen University, Xiamen 361005, China

**Keywords:** neighborhood environment, walking experience, virtual environment, conjoint experiment, latent-class analysis

## Abstract

There is empirical evidence that neighborhood environment characteristics influence individuals’ self-reported affective walking experiences. However, much of the research investigates residents’ affective walking experiences at the neighborhood level using revealed-preference methodologies, making it difficult to identify the separate impacts of characteristics. In addition, empirical studies have not shown enough evidence that individuals from different sociodemographic backgrounds have distinct affective walking experiences. Therefore, the objective of this paper is to explain how different groups of people perceive the characteristics of a neighborhood differently. To do this, this study conducts a conjoint experiment employing videos of virtual environments involving a sample of 295 respondents. Using a latent-class regression model and a multinomial logit model, we are able to determine how individuals and groups perceive neighborhood characteristics differently based on their different emotions. The results somewhat confirmed the findings of the empirical research, indicating that land use mix, connectivity, road size, open space, and greenery are related to a positive walking experience. The level of affective walking experience that individuals associate with neighborhood environmental characteristics is, however, considerably variable. Therefore, our results show that open space and road width are crucial for a walkable neighborhood since they are most helpful to individuals’ subjective well-being.

## 1. Introduction

In car-oriented neighborhoods, environmental issues associated with fossil-fuel use, an increase in societal costs related to traffic congestion, and personal health problems relevant to the widespread use of private automobiles and a sedentary lifestyle are becoming increasingly prevalent [1]. To address these urban issues, cities worldwide are exploring walking-friendly neighborhood environment designs that can encourage more walking and improve individuals’ health and well-being [2]. As a result, individuals’ walking behavior [3], environmental correlates, and benefits to physical and mental health have been studied by a massive body of research, which has developed measuring indicators and scales to assess walkability, walking behavior, neighborhood environment, and well-being [4,5,6,7,8]. Among these, the relationship between neighborhood walking environment and affective walking experience has recently gained increasing scientific interest [9,10].

Empirical studies have provided evidence that neighborhood environment characteristics (e.g., street connectivity, residential density, land-use mix, and traffic safety) influence the self-reported affective walking experience of individuals, and therefore have effects on mental wellbeing [5,11,12,13,14]. On a more individual level, affective experience, as a part of mental wellbeing, is often separated into two components: hedonic—“satisfaction and positive emotions” and eudemonic—“purpose, meaning, or self-actualization” [9,15]. Positive and negative affects—“short-term presence/absence of positive emotions” and cognitive assessment—“long-term satisfaction with life” are frequent components of affective experience [9,16].

Previous research has started to examine the factors that may contribute to the affective experience of walking in general. In many cases, studies use revealed-preference methods, in which respondents assessed various aspects of the neighborhood walking environment using self-rating scales, to investigate residents’ affective experiences on a neighborhood level [17,18,19]. However, because of the strong correlations across neighborhood variables in the revealed-preference data, it is difficult to identify the separate effects of characteristics using this approach [18,19,20,21]. In the field of environmental-preference research, more recently, the conjoint experiment has been increasingly regarded as an alternative research method for increasing resident participation and testing the effects of physical interventions before their actual implementation [1,2,6,22]. In a conjoint experiment, neighborhood characteristics can be varied separately, allowing the separate effects of neighborhood characteristics to be identified by analyzing choices or preference data. However, the use of conjoint experiments to identify the separate effects of neighborhood characteristics on affective walking experience has received only limited attention. Furthermore, empirical studies clearly pointed out that people with different socio-demographic backgrounds have different perceptions of neighborhood environments [23,24,25]. Moreover, whether or not individuals from various socio-demographic backgrounds have different affective walking experiences have received very little attention.

As such, the objective of this research is to answer the following major research question: how do different groups of people perceive the characteristics of the neighborhood differently? Thus, this study will determine the distinct impacts of neighborhood characteristics on the affective walking experience and will provide a clearer explanation for the relationship between socio-demographic characteristics and individual groups. The data for the conjoint experiment are collected through an online survey, which utilizes a video of a virtual environment to represent the neighborhood environment from the perspective of a moving pedestrian. Therefore, the findings should contribute to a better understanding of the relationship between the neighborhood environment and an individual’s affective walking experience.

The rest of this study is organized as follows. The next section discusses the theoretical background of the research on the association between neighborhood characteristics and affective walking experiences. Section 3 discusses the experimental design, data collection, and analysis methods. Section 4 presents the results of the analysis and discussion. Section 5 summarizes the major findings and their policy implications.

## 2. Theoretical Background

This section discusses concepts, measuring instruments, and empirical findings on the influence of the neighborhood environment on the affective walking experience. In particular, we consider the relationship between neighborhood characteristics and components of affective walking experience.

### 2.1. Measuring Affective Walking Experience

Empirical studies proposed that subjective well-being consists of three dimensions: positive affect, negative affect, and cognitive assessment [26]. Among them, positive and negative effects were used to measure the affective walking experience in many cases [13,15,16,27]. Existing studies provide many well-established instruments for retrospectively assessing affective experiences in the past twenty years, the majority of which include Likert-type or semantic differential scales [9,28,29].

In the field of environmental-preference research and transportation planning, Ettema et al. (2010) [28] provided a theoretical framework for quantifying the measurement of subjective well-being in the travel domain. Based on the theoretical framework, Ettema et al. (2011) [30] developed and tested the Satisfaction with Travel Scale (STS), a measure of the affective components of subjective well-being. The STS used a seven-point (−3 to +3) semantic differential scale to assess three components of affective experience (core affect, from negative deactivation to positive activation) [9,31]. The application of STS has been expanded in recent years, and currently includes research from a variety of countries and across a range of travel modes, including commute and non-commute travel [15,32,33,34,35,36]. To further understand the effect of the walking environment on well-being in more detail, Ettema & Smajic (2015) [26] expanded the three components to four items (ranging from −3 to 3 on a scale) to assess the affective walking experience based on the Swedish Core Affect Scale and the STS. These four items were monotone–varied, dull–exciting, unfriendly–friendly, and unsafe–safe [28]. Singleton (2019) [9] and Loo (2021) [14] then consolidated and refined the affective items into multidimensional measures of affective walking experience as follows: happiness, safety, comfort, and annoyance.

However, these studies apply revealed-preference approaches to examine individuals’ affective walking experiences at the neighborhood level, which leads to high correlations between neighborhood characteristics in the dataset [21]. Therefore, it is difficult to discern the different impacts of characteristics using this technique [23,24]. As an alternative approach, the conjoint experiment is gaining popularity, in which neighborhood characteristics can be varied independently, allowing the separate effects of neighborhood characteristics to be determined through the analysis of choice or preference data [1,2,6,23]. Using the conjoint experiment, for example, Birenboim et al. (2021) [20], and van Vliet et al. (2021) [18] have investigated the affective experiences of individuals.

In summary, the existing quantitative research more often used revealed-preference approaches to assess affective effects that inquired about overall satisfaction or preference for walking in general, walking by certain modalities, or walking for diverse purposes [5,9,15]. These classic measurements may partly quantify certain emotional components, but the use of conjoint experiments to determine the different impacts of neighborhood characteristics on the affective walking experience has received less attention and these studies therefore pay less attention to how different groups of people perceive the characteristics of the neighborhood differently [9,37]. In a conjoint experiment, attributes of the alternatives are varied in accordance with a statistical design so that the distinct impacts of the attributes can be established by analyzing choice or preference data [38]. Consequently, it is important and useful to explain the distinct impacts of neighborhood factors on the affective walking experience by using the conjoint experiment.

### 2.2. Neighborhood Environment and Affective Walking Experience

Individual-level examinations of the relationship between neighborhood environment and affective experience have revealed numerous mechanisms via which neighborhood environments can impact affective experiences [15,28,30,31,32,33,34,35,36,37]. Among the most relevant studies are those influencing short-term affective experience through destination-oriented travel walking and activities (e.g., walking for recreation) [9,38].

Empirical studies have clearly pointed out that the neighborhood environment influences the affective walking experience of individuals. Ettema et al. (2015) [26] and Birenboim et al. (2021) [20] pointed out that the neighborhood environment is associated with a sense of happiness and sense of security. Additionally, some studies have found the association between the neighborhood environment and a sense of annoyance [9,24,28].

Some existing studies have further shown that neighborhood factors have positive effects on residents’ affective experiences [22,39,40,41,42,43]. For example, Isaacs (2000) [44] used on-site interviews to discuss the relationship between the aesthetic landscape of neighborhoods and the pedestrian experience. Similarly, Pinder (2005) [45] and Wylie (2009) [46] reported that the landscape (particularly its greenness) influenced pedestrian experiences in a neighborhood. More importantly, many studies investigated the association between neighborhoods’ environmental and contextual characteristics (typically the home neighborhood) and residents’ affective experiences or walking frequency, durations, and distances [22,26,41,44]. Several characteristics are positively correlated with affective experiences, including traffic speed and density, pedestrian safety, street connectivity, residential density, land use mix (accessibility to local facilities), greenery, sidewalks, well-maintained lighting, and cleanliness [15,22,26,38,45,46,47,48,49,50].

Meanwhile, several studies have revealed an inconsistent relationship between socio-economic or demographic characteristics (e.g., gender, ethnicity, educational background, income level, and household structure) and affective walking experience [51,52,53,54,55,56]. One exceptional characteristic is age, since older residents appear to have a more positive effect on walking [9,41,55,57,58]. This may suggest that residents with diverse lifestyles have different walking experiences, so further empirical research is required to confirm this argument.

The existing literature offered measures of individuals’ affective walking experience and demonstrated the association between the neighborhood environment and affective walking experience. Nevertheless, these studies did not explain whether different groups of individuals perceive the characteristics of neighborhood differently. Furthermore, this brief review emphasizes the need for further research utilizing the conjoint experiment to identify the diverse effects of neighborhood environmental characteristics on the affective walking experience of individuals. There is also a need to examine whether individuals from diverse socio-demographic backgrounds have distinct affective walking experiences. However, only a few of the examined studies considered an orthogonal design or a full factorial design for their conjoint experiment, although prior research indicated that a full-fledged experimental design would allow the identification of weights for individual attributes [38]. Therefore, the purpose of the present study is to design a full-fledged experimental design (orthogonal design) for a more rigorous investigation of the relationship between the neighborhood environment and the affective walking experience.

## 3. Methodology

In this section, we present the procedure for designing a conjoint experiment that examines the association between neighborhood environmental characteristics and affective walking experiences. Techniques for data collection and analysis have also been introduced.

### 3.1. The Experimental Design and Survey Instrument

**Figure 1:**
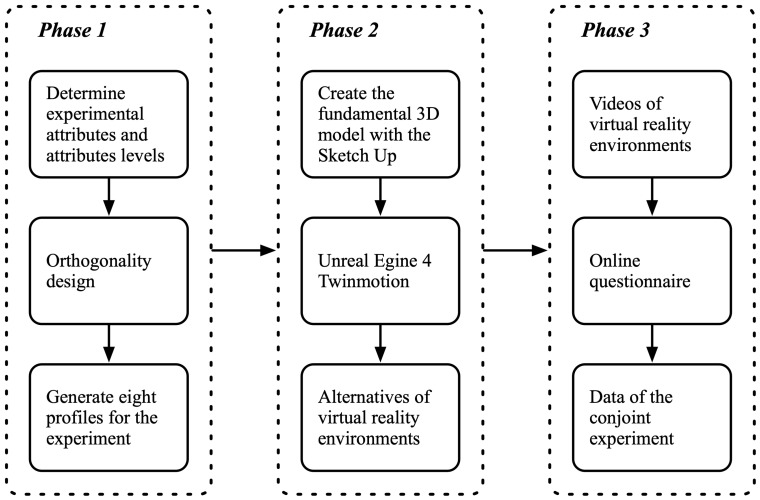
The flowchart of the conjoint experiment design.

The design procedure for the conjoint experiment using a video-based virtual-reality technique consisted of three phases: (1) defining attributes and the levels of the attributes, (2) designing the virtual-reality environment utilizing 3D models, and (3) designing the online survey for participants to reply. Figure 1 depicts the major phases of experiment creation and implementation.

For the definition of the attributes and attribute levels, empirical research demonstrates that land use mix, open space, sidewalks, connectivity, and trees are vital factors in the walking experience [7,17,28,37,52,59]. Therefore, we chose the five aforementioned neighborhood characteristics as design attributes for the experiment. In order to reduce the size of the experimental design, two levels were organized for each attribute to generate alternatives as follows: (1) land use mix: residential only and residential mixed with the commercial area; (2) block connectivity: high and low; (3) road size: two lanes with a narrow pedestrian zone and one lane with a wide pedestrian zone; (4) open space: the street-block with and without open space; and (5) green: the street-block with and without trees. Given this standard, there were 32 (2^5^) potential attribute profiles. However, it was feasible to minimize the number of profiles and avoid attribute correlations. Orthogonality is a mathematical constraint that requires all attributes to be statistically independent of one another so that their effects can be determined by statistical analysis [60]. In this specific case, the whole factorial design can be simplified into an orthogonal design that is made up of eight attribute profiles.

In the next step, we transformed the eight profiles into eight different virtual-reality scenarios by using different visualization software (e.g., SketchUp Pro 2019 and Twinmotion 2019). Table 1 shows how the eight 3D sketch models corresponded to the orthogonal design’s attributes and virtual-reality profiles. Next, we assigned all of the virtual-reality environments a walking viewpoint and then exported them as videos. In order to maintain consistency throughout all of the videos, the walking paths, viewing directions, geographical locations, hours of daylight, seasons, building style, tree color, facilities, and weather all remained the same. Each movie was exactly one minute and thirty seconds long.

In the virtual-reality environments, the questionnaire investigated how the participant felt when he or she watched a video of the virtual environment. Participants rated the virtual environments depending on how they felt. Considering the length of the questionnaire, we displayed four out of the eight virtual-reality videos for each respondent. We asked the participants the degree to which they felt each of the following four feelings: happiness, comfort, annoyance, and security. The questions were posed in the form of statements: “I felt happy/comfortable/annoyed/secure,” and the respondents were asked to rate their feelings on a Likert scale that ranged from completely disagree (1) to completely agree (7) for each item. The survey asked questions about the respondents’ perceptions of the benefits that came from engaging in the virtual environments.

### 3.2. Data Collection and Analysis

Data were collected during the months of April and May 2020, and the respondents for this survey were recruited from a nationwide consumer panel and social media in the Netherlands (Twitter and LinkedIn). The online survey was completed by 308 individuals: 272 from the consumer panel and 36 from social media. To ensure sufficient data quality, respondents who submitted the same answers to each question or completed the VR section in less than eight minutes were eliminated. After data cleaning, the final sample consisted of 295 respondents. and Therefore, 1180 ratings per each item (four virtual watching-trips per respondent) were recorded for analysis. The sample characteristics are shown in Table 2.

Assuming that the dependent variable was roughly of interval level (on the 7-point rating scale), we therefore used regression analysis as the fundamental approach for data analysis. In this case, each affective variable (the four feelings) indicated one dimension of the individuals’ feelings. We utilized the latent-class regression model to identify groups while taking into account the panel structure of the data (repeated observation). The use of the latent-class regression model generates class membership data. In the following step, therefore, the membership data was analyzed using a discrete choice model to identify the association between individuals’ socio-demographic characteristics and their class membership.

The latent-class model is based on the assumption that individuals are implicitly classified into a set of classes, υ, and considers the finite mixture model with V classes of the form [60]:

(1) $$k\left(y|x,\psi \right)=\overset{V}{\underset{\upsilon =1}{\sum }}\pi_{\upsilon }f\left(y|x,\emptyset_{\upsilon }\right)$$

$$\pi_{\upsilon }\geq 0,\;\overset{V}{\underset{\upsilon =1}{\sum }}\pi_{\upsilon }=1$$

where y is a dependent variable with conditional density k, x is a vector of independent variables, $\pi_{\upsilon }$ is the prior probability of class υ, $\emptyset_{\upsilon }$ is the class-specific parameter vector for the density function f, and $\psi ={\left(\pi_{1},\;\ldots ,\;\pi_{V},\;\emptyset_{1},\;\ldots ,\;\emptyset_{V}\right)}^{\prime }$ is the vector of all parameters. According to the model, f is a univariate normal density with class-specific mean $\beta_{\nu }^{\prime }x$ and variance $\sigma_{\nu }^{2}$. We then have $\emptyset_{V}={\left(\beta_{\upsilon }^{\prime },\sigma_{\upsilon }^{2}\right)}^{\prime }$ and Equation (1) describes a latent-class regression model [60,61]. The posterior probability that observation (x,y) belongs to class h is given by [60]:

(2) $$P\left(h|x,y,\psi \right)=\frac{\pi_{h}f\left(y|x,\emptyset_{h}\right)}{\sum_{\upsilon }\pi_{\upsilon }f\left(y|x,\emptyset_{\upsilon }\right)}$$

The posterior probabilities can be utilized to segment data by allocating each observation to the class with the maximum posterior probability. We refer to $f\left(\cdot |\cdot ,\emptyset_{\upsilon }\right)$ as mixture classes, and the groups in the data are induced by these classes [60,62]. The latent class parameters are estimated by the maximum likelihood of a sample of M observations $\left\{\left(x_{1},\;y_{1}),\ldots ,\;(x_{M},\;y_{M}\right)\right\}$, given by [60]:

(3) $$\log L=\overset{M}{\underset{m=1}{\sum }}\log k\left(y_{m}|x_{m},\psi \right)=\overset{M}{\underset{m=1}{\sum }}\log\left(\overset{V}{\underset{\upsilon =1}{\sum }}\pi_{\upsilon }f\left(y_{m}|x_{m},\emptyset_{\upsilon }\right)\right)$$

McFadden’s Rho-square $\left(\rho^{2}=1-LLB/LLO\right)$ is utilized to indicate the goodness-of-fit of the estimated latent-class regression model, and the number of classes V is set by the user [63]. To determine the optimal number of classes, the model estimation was executed several times for various values of V, and the Akaike Information Criterion (AIC=−2(LLB−P)) and Bayesian Information Criterion (BIC=−LLB+[(P/2)∗ln(N)]) were used to determine the optimal number of classes.

In the next step, we used the multinomial logit (MNL) model to find out the relationship between socio-demographic characteristics and class membership. Each individual was assigned to the class with the maximum posterior probability using the posterior probabilities (Equation (2)) [64]. The MNL model is based on the theory of random utility and assumes that $U_{s}$, for an alternative s, is made up of an observable component, $V_{s}$, and a random error component, $\varepsilon_{s}$. The utility of alternative s is given by [62]:

(4) $$U_{s}=V_{s}+\varepsilon_{s}$$

The observable component of utility is typically assumed to be a linear relationship of attribute levels, alternatives, and their corresponding parameters, and the formula is given by:

(5) $$V_{s}=\underset{k}{\sum }\beta_{k}\chi_{sk}$$

where $\chi_{sk}$ represents the value on attribute k of alternative s, and $\beta_{k}$ represents the marginal utility or parameter weight associated with attribute k [62]. The MNL model estimates the probability that alternative s is chosen out of alternatives J, and the model is given by [62,64]:

(6) $$P_{s}=\frac{exp\left(V_{s}\right)}{\sum_{j}^{J}exp\left(V_{j}\right)},\;j=1,\ldots ,\;J$$

where $V_{s}$ is the observable utility component that defined by Equation (5).

The analysis produces four class solutions for each individual as there were four different regression models (four emotions regressed on attributes, respectively).

## 4. Results and Discussion

In this section, the results of the analysis relating to the regression of the four feelings on attributes that varied throughout the experiment are reported. We then discuss the findings from the estimation of the MNL models, which were used to predict class membership based on socio-demographic characteristics.

### 4.1. Results of the Association between Happiness and Environmental Attributes

Firstly, we consider the analysis results of the association between a sense of happiness and the environmental attributes. The statistics of several estimates for the latent-class regression model are shown in Table 3, under the different settings for the number of classes, V. According to the results of the latent-class regression model, the values of the AIC falls as the number of classes grows from one to two classes, but they increase as the number of classes increases to three. As a result, we determined that the first regression model (happiness sense regressed on attributes) should use a number of classes that is equal to the optimal number two. Concerning the effects of attributes on the happiness sense, Table 4 presents the estimation results for the one-class model and the model with two latent classes, in which the ordinary linear regression (one class) model is included for comparison. The adjusted McFadden Rho-square of the latent-class model is considerably higher than the one-class (ordinary linear regression) model, implying strong differences between classes. In addition, we labeled each latent class based on the weights and levels of significance of the independent variables within that class. For instance, the presence of open space carries the most weight for the first class of the latent class model in Table 4, which is named *open space pleasure*.

Considering the estimated findings in Table 4, the one-class model indicates that a mix of commercial and residential land use, a wide pedestrian road, the existence of open space, and the presence of greenery are related to the sense of happiness. The one-class model, however, does not fit the observations well, as is shown by the low value of the adjusted McFadden Rho-square (Adj.Rho^2^ = 0.051). Compared to the one-class model, the adjusted McFadden Rho-square is significantly larger in the two-class model (Adj.Rho^2^ = 0.101), indicating that separating the two classes can capture substantial heterogeneity. In the two-class model, the first class is defined as *open space pleasure* (55.3%), which regards a mix of commercial land use and the existence of open space to be most important for the happiness. The second class is labeled *shop pleasure* (44.7%) because individuals of this group consider a mix of residential with commercial land use to be the most crucial attribute for the sense of happiness. These findings are in line with Diener et al. (2009) [16], Bergstad et al. (2011) [52], and Zhao et al. (2013) [37], who indicate that the sense of happiness was positively associated with the mix of land use and open space.

Moreover, the findings of the MNL model to predict class membership based on socio-demographic characteristics are shown in further detail in Table 5. The first class—*service pleasure*—is considered to be the base category. Individuals in the *shop pleasure* group are less likely to have full-time and high part-time work. As well as having a low or middle income, they are more likely to be immigrants and to reside in detached and semi-detached or terraced houses. Additionally, they are more likely to be living with their children. These findings are partly in line with Ettema et al. (2015) [26] and Lauwers et al. (2021) [13], who pointed out that socio-economic status was associated with a sense of happiness.

### 4.2. Results of the Association between Feeling Comfortable and Environmental Attributes

Next, we discuss the results of the analysis regarding the association between a sense of comfort and environmental attributes. According to Table 6, the AIC and BIC values decrease as the number of classes increases from one to three and then increase again when the number of classes increases from three to four. As a result, the number of classes that define the optimal number for the second regression model (comfortable sense regressed on environmental attributes) is equal to three. Following Table 6, Table 7 presents the estimation results for the three-class model in further detail, along with the comparison results for the one-class model. A wide pedestrian road, the existence of open space, and the presence of greenery are significantly associated with feeling comfortable in the one-class model (Adj.Rho^2^ = 0.027). In comparison to the one-class model, the latent-class model identifies three groups and shows an increase in adjusted McFadden Rho-square (Adj.Rho^2^ = 0.108). The first class, referred to as *shop comfortable* (26.0%), is associated with a mix of commercial with residential land use and the existence of open space. The second class is called *green comfortable* (58.5%) which mainly relates to the existence of open space and the presence of greenery. In addition, a mix of commercial with residential land use also plays a role in this group. The third class is labeled as *space comfortable* (15.5%), where the existence of open space is the dominant factor. These results are somewhat consistent with the findings of Zuniga-Teran et al. (2017) [11], who found an association between neighborhood characteristics and the positive emotions of individuals.

Furthermore, Table 8 presents the estimation results of the MNL model used to predict class membership, with the first class—*shop comfortable*—serving as the reference group. Persons in the *green comfortable* group are more likely to be low-income Dutch because they are more likely to rent semi-detached or terraced houses, and less likely to be middle-earning workers and non-Dutch immigrants. The *space comfortable* group is more likely to have a long-commute time and, as Dutch workers, are more likely to live in detached houses and semi-detached or terraced houses, less likely to have high part-time work, less likely to be low-income and middle-income people, and less likely to own their house. Consequently, we might infer that participants of the *shop comfortable* group are more likely to be non-Dutch immigrants and homeowners.

### 4.3. Results of the Association between Sense of Annoyance and Environmental Attributes

In this section, we present results regarding the relationship between environmental attributes and the sense of annoyance evoked by the environment. As shown in Table 9, the AIC and BIC values fall as the number of classes grows from one to two and rise as the number of classes grows from two to four. The optimal number, therefore, is equal to two for this third regression model (annoyance regressed on the spatial attributes). The detailed estimation results of the third latent-class regression model are shown in Table 10, as well as the results of the one-class model for comparison. The one-class model (Adj.Rho^2^ = 0.025) suggests that residential land use, a narrow pedestrian road, the absence of open space, and the absence of greenery are significantly associated with a sense of annoyance. The latent-class model identifies two groups and has a larger adjusted MacFadden Rho-square (Adj.Rho^2^ = 0.126) than the one-class model. The first class of the latent-class model is labeled as *car annoying* (68.8%), in which a narrow pedestrian road, the absence of open space, and the absence of greenery mainly contribute to the sense of annoyance. It implies that individuals felt annoyed when space was more likely to be occupied by automobiles. The results of the second class show that residential land-use, high connectivity, a narrow pedestrian road, the absence of open space, and the absence of greenery are related to annoyance. Compared to the first class, individuals of the second group likewise felt annoyed when a space demonstrated high connectivity. Hence, the second class is named *connectivity annoying* (31.2%). These findings are consistent with certain other empirical studies [5,15,64], and Loo (2021) [14] revealed that a vehicle-oriented city may lower residents’ positive feelings.

Regarding the results of the estimation of the MNL model, Table 11 offers detailed information for predicting class membership based on socio-demographic characteristics. In the MNL model, the first category, *car annoying*, serves as the reference category. Individuals from the *connectivity annoying* group are more likely to be single, non-Dutch male immigrants who own their houses. They are also less likely to be full-time and highly part-time workers, and less to have long commute time. On the contrary, persons in the *car annoying* group are more likely to be female Dutch, and more likely to rent dwellings.

### 4.4. Results of the Association between Feeling Secure and Environmental Attributes

Finally, we explain the results of the analysis regarding the relationship between the attributes of the environment and the feeling of security evoked by the environment. Table 12 shows that the AIC and BIC values decrease as the number of classes increases from one to two and increase as the number of classes increases from two to four. The best number for the fourth regression model is therefore two, and the model is separated into two subgroups. Table 13 provides the results of the latent two-class regression model, with the one-class model included for comparison. A wide pedestrian road, the existence of open space, and the existence of greenery are associated with a secure sense in the one-class model. However, the one-class model does not fit the data because of the low value of the goodness-of-fit. In contrast to this, the two-class model shows an adjusted McFadden Rho-square (Adj.Rho^2^ = 0.091) that is better than the one-class model (Adj.Rho^2^ = 0.020). The first class is described as *shop safe* (32.5%), in which individuals consider a mix of commercial and residential land use as being the most important attribute related to their feeling of security. A wide pedestrian road and the existence of open space are also associated with the sense of security in this group. The second group is defined as *space safe* (67.5%), because individuals of that group felt more secure on a broad pedestrian road and in an open area. These findings are consistent with that of De Vos et al. (2015) and Herrmann-Lunecke et al. (2021) [64], who indicated that a wide pedestrian sidewalk and open space increase the sense of safety of individuals.

Additionally, Table 14 presents the results of the MNL model which takes *shop safe* as the baseline group. As is indicated in Table 14, participants of the *space safe* group are more likely to be educated, full-time workers, have a long commute, live with a partner, and reside in semi-detached or terraced houses. On the other hand, they are less likely to have a middle income and own their house.

### 4.5. Heterogeneity Findings of the Results

Overall, these findings suggest that there is quite some heterogeneity in the level of affective walking experience that individuals associate with neighborhood environmental attributes. Different environmental attributes contribute to different feelings of individuals. For instance, participants seem to regard open space as the most important component associated with their affective walking experience, because the open space is almost related to all four emotions. The road-size attribute also plays an important role, much of which is contributed to a happiness sense, annoyance sense, and secure sense evoked by the environment. Additionally, land use and greenery are both associated with the happiness sense and annoyance sense, but connectivity is only associated with the annoyance experience.

Moreover, differences in socio-demographic characteristics across groups are very apparent. Regarding the happiness sense, the *open space pleasure* group is comprised of more full-time Dutch employees living in apartments, whereas the *shop pleasure* group consists of more immigrants with their children living in detached and semi-detached or terraced houses. Regarding the comfortable sense, the *shop comfortable* group comprises a greater proportion of immigrants who own their house, while the *green comfortable* group and *space comfortable* group have a greater proportion of Dutch employees who are less likely to own their households. In addition, the *car annoying* group consists of more female Dutch, but the *connectivity annoying* group consists of more single, male immigrants. Lastly, for the secure sense, we found that individuals in the *shop safe* group are more likely to be single homeowners, and individuals in the *space safe* group are more likely to live in a rental residence with others.

## 5. Conclusions

This research aimed to shed light on the relationship between neighborhood environment and affective walking experience, as well as to explain disparities in the affective walking experiences of individuals from diverse socio-demographic backgrounds. To do so, we conducted a conjoint experiment employing videos of virtual environments, allowing participants to virtually walk through and experience the hypothetical neighborhoods in more spatial and social contexts. We then used a dataset from the Netherlands (*n* = 295), estimating four latent-class regression models and four multinomial logit models to address our research objectives.

The results partially accompanied findings from empirical studies suggesting that land use mix, connectivity, road size, open space, and greenery are associated with the affective walking experience. However, the level of affective walking experience that individuals associate with neighborhood environmental attributes is highly heterogeneous. Our findings suggest that open space and road size are critical for a walkable neighborhood because they are mainly beneficial to the subjective well-being of individuals.

Regarding the relationship between an individual’s affective walking experiences and their sociodemographic characteristics, we find that individuals with different sociodemographic backgrounds perceive different emotions in response to environmental attributes. For example, full-time Dutch workers who live in apartments are more likely to feel happiness in an open space, whereas single, male immigrants are more likely to feel annoyance in a high-connectivity street. Therefore, these findings might imply that a walkable neighborhood design must consider different socio-economic statuses and cultural backgrounds in different neighborhoods.

The findings of this study have critical implications for urban design policy making. First, in line with the previous findings, we recommend that urban design guidelines should require more open spaces, which were the most important component associated with individuals’ affective walking experiences in the neighborhood, in order to bring about an increased sense of happiness, comfort, and security. Second, this study indicates that urban design policies should encourage wider pedestrian zones in neighborhoods to improve the neighborhood walkability as well as the subjective well-being of individuals. Third, and most importantly, we suggest that, prior to neighborhood planning, designers should conduct detailed research on the socio-economic status and cultural background of residents, including family size and job category, etc. This will allow designers to provide targeted designs based on the heterogeneity of residents and to better enhance the walkability of the neighborhood.

Although these results are interesting and meaningful, there are still several limitations that might be addressed in future studies. First, our experiment and data analysis focused on the typical Dutch setting and interpreted only five neighborhood environmental attributes in detail. Our results and findings, therefore, are more useful and meaningful for the built environment in the Dutch context. In light of the fact that regional variances are so significant, future research should take into account different types of geographical and cultural settings with different attributes. Regarding the second point, we utilized an online video presentation in our experiment, but a VR headset and lab equipment would have provided a more realistic and immersive experience for participants [18,38]. Lastly, we reflect on the fixed path and viewing orientation of our video representation. In a more realistic virtual environment, respondents would be able to walk around and design their own paths. As a result, the experiment developed in this research would need be repeated in the lab utilizing full-fledged virtual-reality (VR) technology. Respondents would then be able to walk and look around more randomly in the virtual-reality environment. Addressing these issues will increase our understanding of the relationship between the neighborhood environment and subjective mental well-being.

## Figures and Tables

**Table 1:** The design profiles of the conjoint experiment.

Profiles		Attributes Levels	Virtual Environments
Profile 1	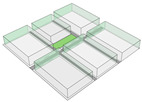	Residential land use High connectivity Two lanes with a narrow pedestrian zone Has open space in the block Has trees in the block	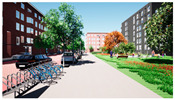
Profile 2	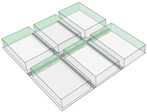	Residential land use High connectivity Two lanes with a narrow pedestrian zone Does not have open space in the block Does not have trees in the block	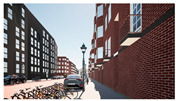
Profile 3	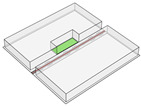	Residential land use Low connectivity One lane with a wide pedestrian zone Has open space in the block Does not have trees in the block	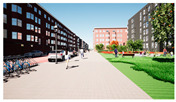
Profile 4	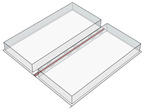	Residential land use Low connectivity One lane with a wide pedestrian zone Does not have open space in the block Has trees in the block	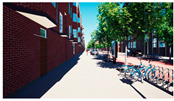
Profile 5	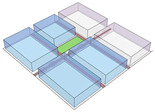	Mixed with commercial area High connectivity One lane with a wide pedestrian zone Has open space in the block Does not have trees in the block	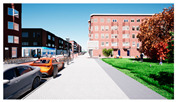
Profile 6	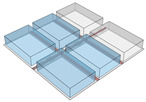	Mixed with commercial area High connectivity One lane with wide pedestrian zone Does not have open space in the block Has trees in the block	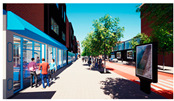
Profile 7	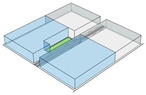	Mixed with commercial area Low connectivity Two lanes with a narrow pedestrian zone Has open space in the block Has trees in the block	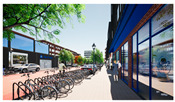
Profile 8	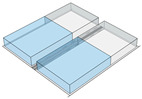	Mixed with commercial area Low connectivity Two lanes with a narrow pedestrian zone Does not have open space in the block Does not have trees in the block	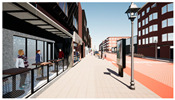

**Table 2:** The sample characteristics.

	Sample (*n*)	Sample (%)
***Age (Years)***	295	43.64 (average years)
***Gender***		
Male	131	44.4
Female	164	55.6
***Education***		
Primary	8	2.70
Medium	133	45.1
High (BSc or higher)	154	52.2
***Work status***		
Full time work	117	39.7
Part-time work (high, 21–37 h)	67	22.7
Part-time work (low, 1–21 h)	26	8.80
No paid work	85	28.8
***Travel time for work***		
Low commute time	138	46.8
Medium commute time	58	19.6
Long commute time	15	5.10
Others	84	28.5
***Gross income (per year)***		
Income under neighborhood average	73	24.7
Income same as neighborhood average	107	36.3
Income above neighborhood average	77	26.1
Others (I don’t want to answer)	38	12.9
***Ethnic background***		
Dutch	280	94.9
Non-Dutch	15	5.10
***Household type***		
Single	71	24.1
Couple without child(ren)	102	34.6
Parents	91	30.8
Others (I don’t want to answer)	31	10.5
***Dwelling type***		
Detached house	46	15.6
Semi-detached or terraced house	160	54.3
Apartment	75	25.4
Other dwelling type	14	4.70
***Houseowner situation***		
Owns house	197	66.8
Rents house	98	33.2
**Total**	**295 respondents**	**100**
	**1180 rating**	

**Table 3:** Statistics for latent-class regression models (*happiness sense*).

No. of Class	Parameters	Log Likelihood Function	AIC	BIC
1	7	−2143.89	4301.77	4337.29
2	15	−2121.24	4272.49	4348.59
3	23	−2120.27	4286.54	4403.23
4	31	−2102.75	4266.18	4423.45

Note: AIC = Akaike information criterion; BIC = Bayesian information criterion.

**Table 4:** Results for latent-class regression models (*happiness sense*).

		Two Latent Classes Model	
	One-Class Model	Open Space Pleasure	Shop Pleasure
	Estimate	Estimate	Estimate
Residential area	−0.242 ***	−0.356 ***	−0.661 ***
High block connectivity	−0.110	−0.152	−0.028
Two lanes with a narrow pedestrian size	−0.328 ***	−0.167 *	−0.275 **
Has open space in the street	0.393 ***	0.414 ***	0.309 ***
Has trees in the street	0.167 **	0.171 *	0.332 ***
Share of the individuals	100%	55.3%	44.7%
McFadden’s Rho-squared:	0.055	0.107	
Adjusted McFadden’s Rho-squared:	0.051	0.101	

Note: ***, **, * ==> Significance at 1%, 5%, 10% level.

**Table 5:** Results of the MNL models (*happiness sense*).

	Shop Pleasure
	Coefficients
***Work status***	
Full time work	−0.680 ***
Part-time work (high, 21–37 h)	−1.398 ***
No paid work (reference)	/
***Gross income (per year)***	
Income under neighborhood average	−1.027 ***
Income same as neighborhood average	−0.680 ***
Income above neighborhood average (reference)	/
***Ethnic background***	
Non-Dutch	0.694 ***
Dutch (reference)	/
***Household type***	
Couple without child(ren)	−0.720 ***
Single (reference)	/
***Dwelling type***	
Detached house	0.904 ***
Semi-detached or terraced house	0.422 **
Other dwelling type	−0.724 **
Apartment (reference)	/

Note: ***, **, ==> Significance at 1%, 5% level.

**Table 6:** Statistics for latent-class regression models (*comfortable sense*).

No. of Class	Parameters	Log Likelihood Function	AIC	BIC
1	7	−2081.31	4176.61	4212.12
2	15	−2054.21	4138.43	4214.53
3	23	−2049.24	4127.23	4213.92
4	31	−2033.39	4128.79	4286.06

Note: AIC = Akaike information criterion; BIC = Bayesian information criterion.

**Table 7:** Results for latent-class regression models (*comfortable sense*).

	One-Class Model	Shop Comfortable	Green Comfortable	Space Comfortable
	Estimate	Estimate	Estimate	Estimate
Residential area	−0.096	−0.215 **	−0.034	−0.202
High block connectivity	−0.077	−0.152	−0.032	−0.244
Two lanes with a narrow pedestrian size	−0.259 ***	−0.141	−0.257 ***	−0.241
Has open space in the street	0.368 ***	0.171 *	0.495 ***	0.378 **
Has trees in the street	0.164 **	0.039	0.251 ***	0.964
Share of the individuals	100%	26.0%	58.5%	15.5%
McFadden’s Rho-squared:	0.031	0.119		
Adjusted McFadden’s Rho-squared:	0.027	0.108		

Note: ***, **, * ==> Significance at 1%, 5%, 10% level.

**Table 8:** Results of the MNL models (*comfortable sense*).

	Green Comfortable	Space Comfortable
	Coefficients	Coefficients
Age		0.025 ***
***Work status***		
Part-time work (high, 21–37 h)		−1.181 ***
No paid work (reference)	/	/
***Travel time for work***		
Long commute time		1.969 ***
Low commute time (reference)		
***Gross income (per year)***		
Income under neighborhood average		−0.823 **
Income same as neighborhood average	−0.573 ***	−0.678 **
Others (I don’t want to answer)		0.767 **
Income above neighborhood average (reference)	/	/
***Ethnic background***		
Non-Dutch	−0.708 **	−0.868 *
Dutch (reference)	/	/
***Dwelling type***		
Detached house		0.778 **
Semi-detached or terraced house	0.720 ***	1.408 ***
Apartment (reference)	/	/
***House-owner situation***		
Owns house	−0.370 **	−0.781 ***
Rents house (reference)	/	/

Note: ***, **, * ==> Significance at 1%, 5%, 10% level.

**Table 9:** Statistics for latent-class regression models (*annoyance sense*).

No. of Class	Parameters	Log Likelihood Function	AIC	BIC
1	7	−2205.11	4424.23	4459.74
2	15	−2100.15	4220.29	4306.39
3	23	−2088.86	4223.72	4340.41
4	31	−2085.79	4233.59	4390.86

Note: AIC = Akaike information criterion; BIC = Bayesian information criterion.

**Table 10:** Results for latent-class regression models (*annoyance sense*).

		Two Latent Classes Model	
	One-Class Model	Car Annoying	Connectivity Annoying
	Estimate	Estimate	Estimate
Residential area	0.277 ***	0.278 **	0.213 **
High block connectivity	0.121	−0.059	0.143 **
Two lanes with a narrow pedestrian size	0.214 **	0.228 **	0.169 **
Has open space in the street	−0.231 **	−0.305 ***	−0.139 *
Has trees in the street	−0.295 ***	−0.324 ***	−0.173 **
Share of the individuals	100%	68.8%	31.2%
McFadden’s Rho-squared:	0.029	0.133	
Adjusted McFadden’s Rho-squared:	0.025	0.126	

Note: ***, **, * ==> Significance at 1%, 5%, 10% level.

**Table 11:** Results of the MNL models (*annoyance sense*).

	Connectivity Annoying
	Coefficients
***Gender***	
Female	−0.237 *
Male (reference)	/
***Work status***	
Full time work	−0.681 ***
Part-time work (high, 21–37 h)	−0.610 ***
No paid work (reference)	/
***Travel time for work***	
Long commute time	−2.256 ***
Low commute time (reference)	/
***Gross income (per year)***	
Others (I don’t want to answer)	0.851 ***
Income above neighborhood average (reference)	
***Ethnic background***	
Non-Dutch	1.085 ***
Dutch (reference)	/
***Household type***	
Couple without child(ren)	−0.674 ***
Parents	−0.805 ***
Others (I don’t want to answer)	−0.451 *
Single (reference)	/
***House-owner situation***	
Owns house	0.905 ***
Rents house (reference)	/

Note: ***, * ==> Significance at 1%, 10% level.

**Table 12:** Statistics for latent-class regression models (*secure sense*).

No. of Class	Parameters	Log Likelihood Function	AIC	BIC
1	7	−1975.16	3964.32	3999.84
2	15	−1961.39	3932.77	4028.87
3	23	−1947.98	3941.96	4058.65
4	31	−1935.77	3933.54	4090.81

Note: AIC = Akaike information criterion; BIC = Bayesian information criterion.

**Table 13:** Results for latent-class regression models (*secure sense*).

		Two Latent Classes Model	
	One-Class Model	Shop Safe	Space Safe
	Estimate	Estimate	Estimate
Residential area	−0.052	−0.226 **	0.015
High block connectivity	−0.062	−0.116	−0.017
Two lanes with a narrow pedestrian size	−0.184 **	−0.188 **	−0.218 ***
Has open space in the street	0.273 ***	0.157	0.296 ***
Has trees in the street	0.129 *	0.199 **	0.112
Share of the individuals	100%	32.5%	67.5%
McFadden’s Rho-squared:	0.020	0.091	
Adjusted McFadden’s Rho-squared:	0.016	0.083	

Note: ***, **, * ==> Significance at 1%, 5%, 10% level.

**Table 14:** Results of the MNL models (*secure sense*).

	Space Safe
	Coefficients
Age	0.013 **
***Education***	
Medium	1.430 ***
High (BSc or higher)	1.393 ***
Primary (reference)	/
***Work status***	
Full time work	0.821 ***
No paid work (reference)	/
***Travel time for work***	
Long commute time	0.948 **
Low commute time (reference)	
***Gross income (per year)***	
Income same as neighborhood average	−0.719 ***
Income above neighborhood average (reference)	
***Household type***	
Couple without child(ren)	0.826 ***
Parents	0.694 ***
Others (I don’t want to answer)	0.969 ***
Single (reference)	/
***Dwelling type***	
Semi-detached or terraced house	0.604 ***
Others dwelling type	−0.863 ***
Apartment (reference)	/
***House-owner situation***	
Owns house	−1.153 ***
Rents house (reference)	

Note: ***, **, => Significance at 1%, 5% level.
